# Introduction of Deep Learning-Based Infrared Image Analysis to Marginal Reflex Distance1 Measurement Method to Simultaneously Capture Images and Compute Results: Clinical Validation Study

**DOI:** 10.3390/jcm12237466

**Published:** 2023-12-01

**Authors:** Bokeun Song, Hyeokjae Kwon, Sunje Kim, Yooseok Ha, Sang-Ha Oh, Seung-Han Song

**Affiliations:** 1 Department of Plastic and Reconstructive Surgery, Chungnam National University Hospital, Daejeon 35015, Republic of Korea; bogenlove@gmail.com (B.S.); kwon.hyeokjae@cnuh.co.kr (H.K.); kkk9243@naver.com (S.K.); useok4u@naver.com (Y.H.); djplastic4073@gmail.com (S.-H.O.); 2 Department of Medical Science, College of Medicine, Chungnam National University, Daejeon 35015, Republic of Korea; 3 Department of Plastic and Reconstructive Surgery, College of Medicine, Chungnam National University, Daejeon 35015, Republic of Korea

**Keywords:** blepharoplasty, deep learning, machine learning, eye movement measurements

## Abstract

Marginal reflex distance1 (MRD1) is a crucial clinical tool used to evaluate the position of the eyelid margin in relation to the cornea. Traditionally, this assessment has been conducted manually by plastic surgeons, ophthalmologists, or trained technicians. However, with the advancements in artificial intelligence (AI) technology, there is a growing interest in the development of automated systems capable of accurately measuring MRD1. In this context, we introduce novel MRD1 measurement methods based on deep learning algorithms that can simultaneously capture images and compute the results. This prospective observational study involved 154 eyes of 77 patients aged over 18 years who visited Chungnam National University Hospital between 1 January 2023 and 29 July 2023. We collected four different MRD1 datasets from patients using three distinct measurement methods, each tailored to the individual patient. The mean MRD1 values, measured through the manual method using a penlight, the deep learning method, ImageJ analysis from RGB eye images, and ImageJ analysis from IR eye images in 56 eyes of 28 patients, were 2.64 ± 1.04 mm, 2.85 ± 1.07 mm, 2.78 ± 1.08 mm, and 3.07 ± 0.95 mm, respectively. Notably, the strongest agreement was observed between MRD1_deep learning (DL) and MRD1_IR (0.822, *p* < 0.01). In a Bland–Altman plot, the smallest difference was observed between MRD1_DL and MRD1_IR ImageJ, with a mean difference of 0.0611 and ΔLOA (limits of agreement) of 2.5162, which was the smallest among all of the groups. In conclusion, this novel MRD1 measurement method, based on an IR camera and deep learning, demonstrates statistical significance and can be readily applied in clinical settings.

## 1. Introduction

**Figure 1:**
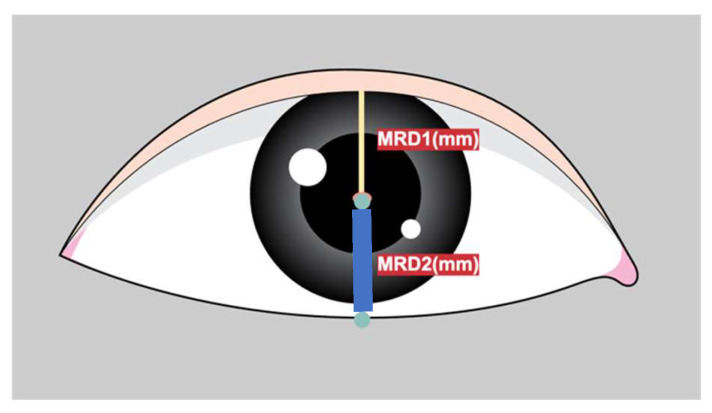
Figure depicts the definition of MRD1 (distance between pupillary light reflex and upper eyelid margin in millimeters) and MRD2 (distance between pupillary light reflex and the lower lid margin in millimeters).

Marginal reflex distance1 (MRD1) is crucial for the evaluation and management of ptosis, a condition in which the upper eyelid droops over the eye [1]. MRD1 is the distance between the center of the pupillary light reflex and the upper eyelid margin with the eye in primary gaze (Figure 1).

Ptosis severity is categorized as follows based on MRD1 values: mild (3–4 mm), moderate (2–3 mm), or severe (0–2 mm). MRD1 measurement is fundamental to patient assessment and surgery choice in facial and ophthalmic plastic surgery [2]. The manual measurement of MRD1 is labor-intensive, subjective, and highly susceptible to human error [1]. Also, the direct MRD1 measurement method using a ruler is neither accurate nor repeatable, and even time consuming. To overcome these limitations, several studies have attempted to automate MRD1 measurements.

Image analysis software for MRD1 and other periorbital measurements from patient photographs has been previously introduced [3,4].

These digital photography methods have the advantage of being more scientific, objective, and reproducible than traditional manual methods. However, the disadvantage of these digital software is that clinicians still need to assess the imported images. In other words, these previously introduced methods are semi-automated in that they rely on significant user and computer interaction after image acquisition and depend on the observer to identify edges and facial morphological features.

AI has revolutionized medical imaging by leveraging its powerful algorithms to analyze complex datasets, leading to a more accurate and efficient diagnosis, improved image interpretation, and enhanced patient care. Deep learning algorithms are effective at recognizing meaningful patterns in images and extracting specific features from medical images. Convolutional neural network (CNN)-based deep learning methods, a subset of machine learning techniques, have been state-of-the-art in AI for years, leading to enhanced performance in various medical applications. With the development of artificial intelligence, there are many different methods being introduced to overcome these semi-automated methods and provide more systematic and accurate measurements [5,6,7,8,9].

However, there are few studies that introduce artificial intelligence technologies based on deep learning that measure MRD1.

**Figure 2:**
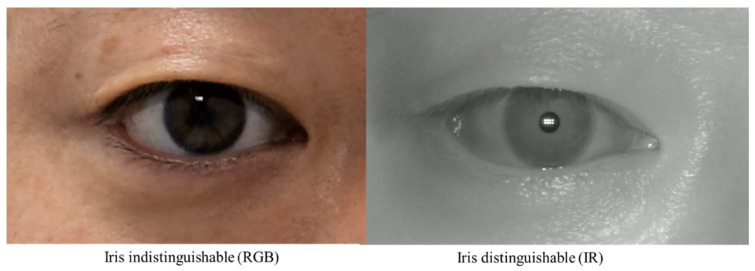
Two Images were taken from the same patient. RGB Image: circumferential boundaries and centers of pupils were indistinguishable from the nearby iris (**right**). IR Image: circumferential boundaries and centers of pupils were distinguishable from the nearby iris (**left**). RGB, red-blue-green; IR, infrared.

While these studies have the advantage of applying artificial intelligence technology to MRD1, they have some limitations. First, there is a limitation in that the acquired images are all digital images based on DSLR cameras. This is because MRD1, by definition, needs to find the center of the pupil, and these RGB-based images have difficulty distinguishing between the pupil and iris in people with dark irises (Figure 2). Second, as with the semi-automated methods mentioned earlier, there is still a disconnect between the photometry and the calculation of the MRD1 results. These points have been an obstacle to application in clinical practice.

To overcome the limitations mentioned above, here, we introduce a novel IR image-based deep learning-assisted integrated MRD1 measurement method. Using the visible light spectrum to examine the characteristics of the pupil is markedly limited in patients with dark-colored irises [9,10]. Also, prior studies revealed that even in controlled lighting conditions with various approaches, it is extremely difficult to segment the pupil from a dark brown iris [10,11]. To overcome the obstacles of measuring MRD1, we introduce an infrared (IR)-image-based deep learning model combined with a measurement device that simultaneously collects and computes the data. This is because the IR image accurately distinguishes between the iris and pupil, and developing a deep learning model utilizing this image will result in higher accuracy.

To the best of our knowledge, there have been no previous studies using IR imaging for MRD1 measurement and no automated AI models for MRD1 measurement have been reported in combination with measurement devices.

This study aimed to determine the efficiency and accuracy of a real-time IR image-based deep learning algorithm compared to existing methods.

## 2. Materials and Methods

### 2.1. Deep Learning Model Selection—RITnet

Several algorithms for pupil center detection were originally used for gaze tracking. In this study, we used the RIT net model. RITnet is an eye semantic segmentation model based on deep learning. It is a deep neural network that combines U-net and DenseNet to overcome the difficult problems of existing eye semantic segmentation models, such as detection accuracy and robustness according to each person, real-time processing, and unrestricted lighting environments. RITnet has shown good performance in the open eye dataset (OpenEDS) semantic segmentation challenge, achieving an accuracy of 95.3%; therefore, it was selected and utilized as a suitable deep learning model for our pupil detection model of MRD1 measurements [12].

RITnet covers the area of interest in close-up images of the human eye and is a model that segments the pupil, iris, and eyelid in the human eye.

**Figure 3:**
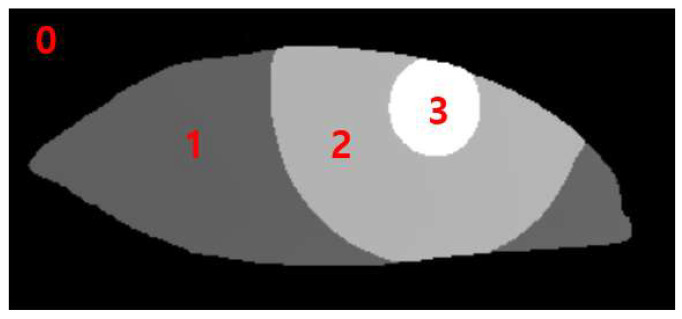
0: background border; 1: sclera; 2: iris; 3: pupil.

The data output from the RITnet model is classified into four different categories, where each pixel is classified as background, iris, pupil, or eye. The classification of each pixel into a trained class is a typical result of semantic segmentation. To measure MRD1, pupil data were used to detect the pupil circle, as in Figure 3 (3). The data for the upper eyelid from Figure 3 (1) measured MRD1 by utilizing the background border at 0 (Figure 3).

### 2.2. Specific Information about Platform Configuration

**Figure 4:**
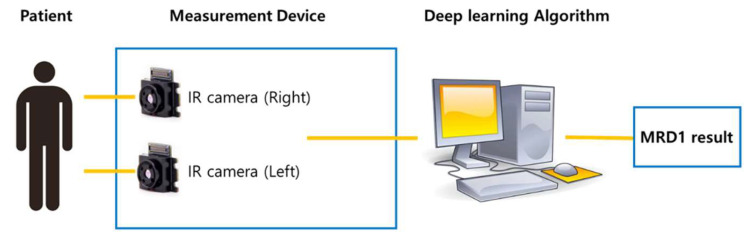
Schematic diagram of newly developed MRD1 measurement platform. Note that MRD1 result can be obtained directly via image taken by IR camera.

Two IR cameras, each measuring one eye, were installed in the hardware device, and deep learning software algorithms were installed on the computer. The hardware device was recognized as one capable of simultaneously capturing an image and computing the algorithm and MRD1 measurement results (Figure 4).

Specific information about hardware subunits.
Laptop computer specification
OS: Microsoft windows 10
CPU: Intel^®^ Core™ i5-1240F
RAM: DDR 16G
Storage: 10TB
GPU: NVIDA GeForce RTX 4090

### 2.3. Image Analysis

This prospective observational study included 154 eyes from 77 patients aged over 18 years, who visited Chungnam National University Hospital between 1 January 2023, and 29 July 2023. This study adhered to the principles of the Declaration of Helsinki. Informed consent was obtained for the publication of patients’ images both online and in print media. This study received approval from the Ethics Committee of Chungnam National University Hospital, Daejeon, Korea (CNUH-IRB No. 2023-06-098). Patient demographics, including age, sex, and history of plastic surgery, were assessed. The mean age of the patients was 47.18 with a standard deviation of 14.65. A total of 46 patients were female, and 31 patients were male. Forty-six patients received blepharoplasty surgery after the image was taken. Twelve patients received surgery before the image was taken.

Four different MRD1 datasets were collected from patients using three different measurement methods, each unique to the patients.

1. Traditional manual MRD1 measurements were obtained using a penlight by three different clinicians (MRD1_manual). The average of the three values obtained above is called MRD1_manual.
2. Bilateral primary gaze photographs were taken using a Nikon D7500 digital camera (Nikon, Tokyo, Japan), and MRD1 images were analyzed using ImageJ software (version 1.53t) with the Java platform version (MRD1_RGB_ImageJ). To compensate for the time and position changes being measured, we positioned the DSLR camera at the same distance as the IR camera and in the same position (same head fixator) and measured the images. ImageJ analysis was performed using an IR camera image (MRD1_IR_ImageJ). We also took 10 consecutive photographs during the same time period and averaged their values to obtain the data.
3. ImageJ analysis was performed using an IR camera image (MRD1_IR_ImageJ).
4. Novel MRD1 measurements using an IR camera image and a deep learning method (RIT-net) were introduced in this study (MRD1_DL).

First, a penlight was used to illuminate the cornea, and the clinician fixated the brow on the side being measured or lifted the brow on the opposite side so that the patient did not raise the ptotic lid. The corneal light reflex was observed, and the distance between the cornea and the upper lid margin was recorded.

Second, frontal facial photographs were taken with the eyes in the primary gaze position, with the patient sitting. A Nikon D7500 digital camera (Nikkon corportation, Tokyo, Japan) was used for the light. Digital images were transferred to a personal computer and analyzed using ImageJ software (MRD1_RGB_ImageJ).

Third, a 5 mm circle was placed on the lower lid as a reference. The scale was only necessary for gold-standard measurements and not for the deep learning model or determining the accuracy of the model. Bilateral photographs of each patient were captured using the IR camera installed in the hardware and exported. Images exported from the computer were analyzed for comparison using ImageJ software (MRD1_IR_ImageJ).

**Figure 5:**
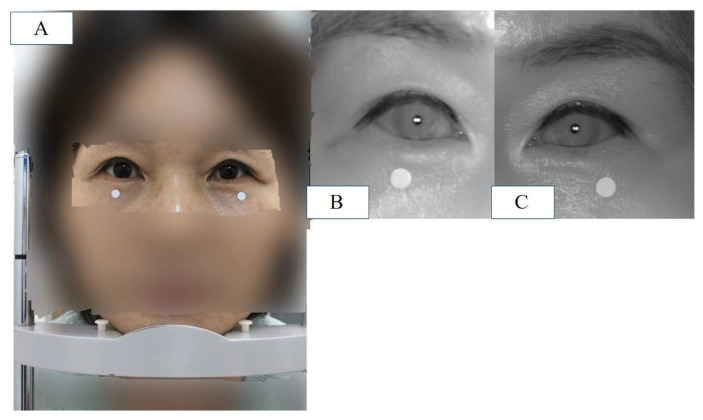
(**A**) Patient is placed in front of the device and instructed to keep eyes in primary gaze in a sitting position, and a chin fixator is used (MRD1_RGB). (**B**) IR image of patient’s right eye in the same position. (**C**) IR image of patient’s left eye in the same position. Note that the white dot in the figure is 5mm in diameter.

Finally, the newly developed IR camera images were captured with the eyes in primary gaze and the participant in a sitting position. Before the measurements were taken, calibration was performed to ensure the accuracy of the reference board. The measured MRD1 values were displayed in the computer software and collected (MRD1_DL) (Figure 5).

The exclusion criteria for this study are as follows.

Exclusion criteria:

(1) Data were excluded if MRD1 values were not measured by three independent clinicians (MRD1_manual).
(2) Data with manually measured MRD1 values of 0 or negative values were excluded (MRD1_manual).
(3) Data where the boundary between pupil and iris was not clearly distinguishable in the DLSR image were excluded (MRD1_RGB_ImageJ).
(4) The average MRD1 values measured via the manual method and those with a difference of more than 1.0 mm from the reference value (MRD1_RGB_ImageJ or MRD1_IR_ImageJ) were excluded from the data for reliability reasons.
(5) Data for which MRD1 values were not obtained by the machine were excluded (MRD1_DL).
(6) The patients who underwent any blepharoplasty surgery within 1 month were excluded.

Under these criteria, 56 eyelids from 28 patients were included, while 98 eyelids from 49 patients were excluded. The mean age of the patients included in this study was 42.48 with a standard deviation of 17.6. A total of 16 patients were female and 12 patients were male. Twenty-two patients received blepharoplasty surgery after the image was taken.

### 2.4. Statistical Analysis

The mean value of each MRD1 measurement and the difference between them were analyzed using a one-way analysis of variance (ANOVA) test. A box-plot graph was plotted to visually describe each different measurement. The correlation between the two measurements was calculated using Pearson’s correlation. Linear regression and Bland–Altman plot analyses were performed to statistically examine the agreement between the different measurement methods. Statistical significance was set at *p* < 0.05. Data obtained from the study were analyzed using the Statistical Package for the Social Sciences version 26 (IBM Corporation, Armonk, NY, USA).

## 3. Results

**Figure 6:**
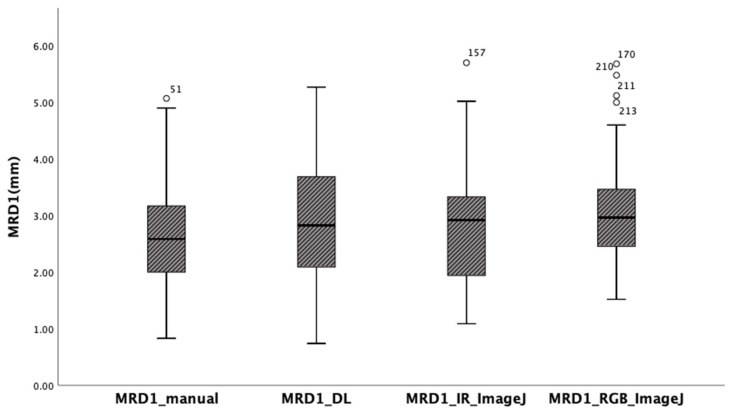
Box-plot analysis showing four different MRD1 measurements. Note that MRD1_DL and MRD1_IR_ImageJ show the strongest correlation.

The mean of MRD1 values measured using the manual method with a penlight, deep learning method, ImageJ analysis from RGB eye images, and ImageJ analysis from IR eye images in 56 eyes of 28 patients were 2.64 ± 1.04 mm, 2.85 ± 1.07 mm, 2.78 ± 1.08 mm, and 3.07 ± 0.95mm, respectively. The box-plot analysis shows the strongest correlation between the MRD1_and MRD1_IR_ImageJ groups (Table 1) (Figure 6).

The ANOVA test indicated that there is no statistically significant difference between the four different methods. (*p* = 0.168) (Table 2). In the post hoc analysis, differences were observed among the four groups, but they were not statistically significant (Table 3).

Pearson’s correlation values were calculated for the four different results measured using the different methods to determine their correlation with the gold standard. In this study, we assumed that MRD1 measured using ImageJ is the gold standard. In other words, we set both MRD1_RGB_ImageJ and MRD1_IR_ImageJ as the gold standard. The correlation coefficient between MRD1_manual and MRD1_RGB ImageJ was 0.656, and that between the MRD1_manual method and MRD1_IR ImageJ was 0.603. Also, the correlation between MRD1_DL and MRD1_RGB_ImageJ was 0.543, and that between MRD1_and MRD1_IR ImageJ was 0.822. Notably, the highest correlation coefficient among different measurement methods was observed between MRD1_DL and MRD1_IR_ImageJ. The highest correlations were observed in the MRD1_DL and MRD1_IR_ImageJ groups (Pearson correlation: 0.822, *p* < 0.01) (Table 4).

**Figure 7:**
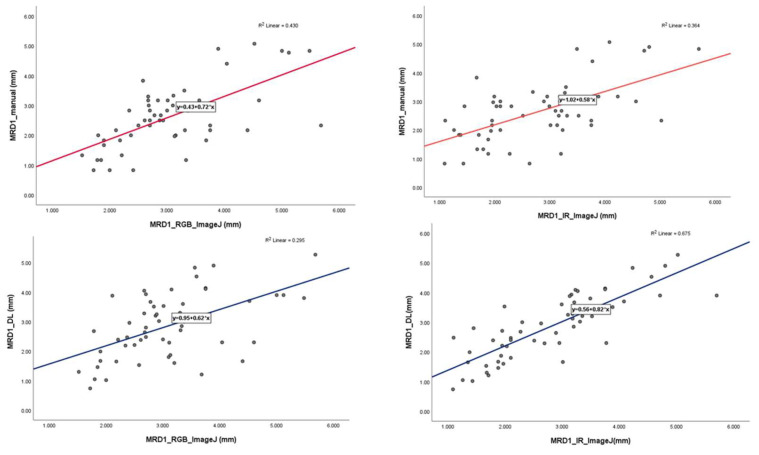
Linear regression analysis with scatter-plot diagram showing correlations between different methods.

**Figure 8:**
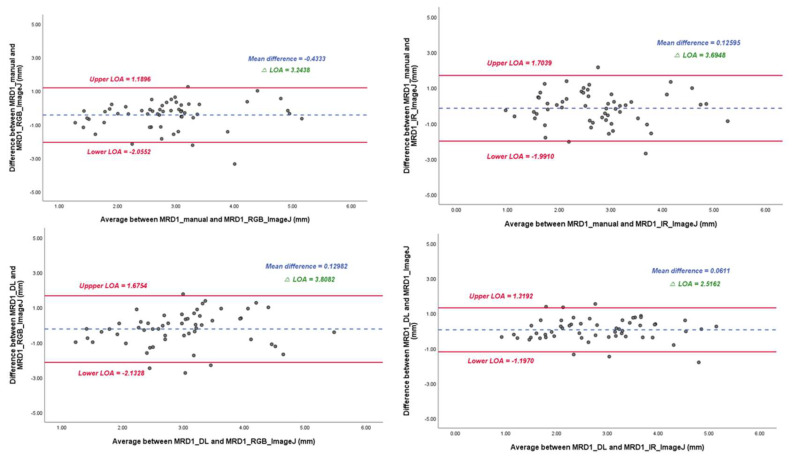
Bland–Altman plot results of MRD1 results between four different methods. ΔLOA: difference between the upper and lower limits of agreement. Note that mean difference and ΔLOA of MRD1_DL and MRD1_IR_ImageJ are 0.061 and 2.5162, respectively.

In the linear regression model, the slope of MRD1_manual and MRD1_RGB ImageJ, with which most of the studies were compared, was 0.72 with an R^2^ value of 0.430. However, the slopes of MRD1_manual and MRD1_IR ImageJ and MRD1_DL and MRD1_IR ImageJ were 0.58 with an R^2^ value of 0.364 and 0.82 with an R^2^ value of 0.675, respectively (Figure 7). For a more precise comparison, we constructed a Bland–Altman plot to determine the degree of agreement between the different methods. The mean differences between MRD1_manual and MRD1_RGB ImageJ, MRD1_manual and MRD1_IR ImageJ, MRD1_DL and MRD1_RGB ImageJ, and MRD1_DL and MRD1_IR ImageJ were −0.4333, 0.1260, 0.1298, and 0.0611, respectively. The differences between the upper and lower limits of agreement were 3.2438, 3.6948, 3.8082, and 2.5162, respectively. The smallest difference was observed between MRD1_DL and MRD1_IR ImageJ (2.5162, *p* < 0.05) (Figure 8).

### Real Patient Data

**Figure 9:**
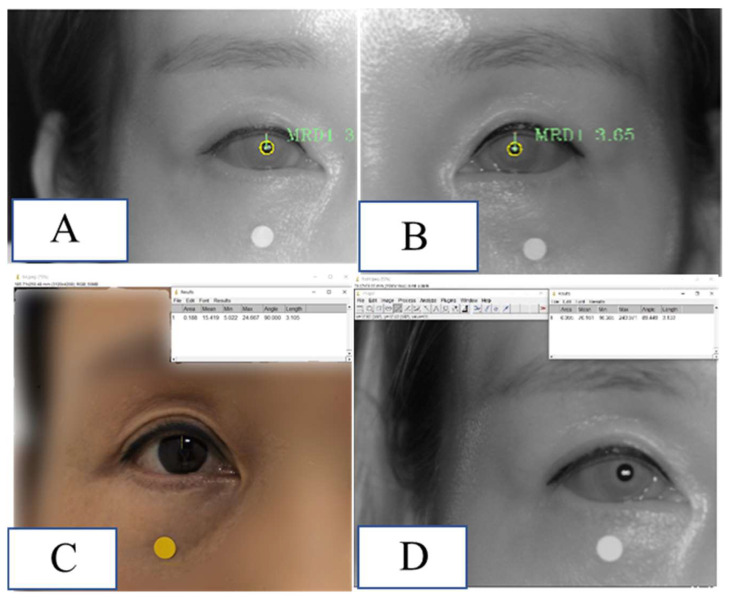
Demonstration of MRD1 measured via the method used in this study in a real patient. (**A**) MRD1 of right eye measured via MRD1_DL was 3.06 mm. (**B**) MRD1 of left eye measured via MRD1_DL was 3.65 mm. (**C**) MRD1 of right eye measured via MRD1_RGB_ImageJ was 3.10 mm. (**D**) MRD1 of right eye measured via MRD1_IR_ImageJ was 3.13 mm. Note that the white and dot in the figure is 5mm in diameter.

A 43-year-old woman visited an outpatient clinic and had her MRD1 measured using the method proposed in this study. MRD1 was measured by the three methods proposed in this study and was taken in a similar time and position. The difference between MRD1_RGB_ImageJ and MRD1_IR_ImageJ compared to MRD1_DL was 0.04 mm and 0.07 mm, respectively (Figure 9).

## 4. Discussion

Manual measurements of MRD1 and MRD2 are used for the clinical evaluation of ptosis and the surgical planning for ptosis repair [13]. However, the existing manual measurement method using a penlight is limited by interobserver variability, reproducibility, patient movement, and poor cooperation with testing, presenting a challenge, and a more precise and scientific measurement method is required [13].

Furthermore, small variations may impact clinical value, research, and audit value, which require a more precise and accurate method. The computer-assisted analysis of facial photographs for the measurement of MRD1, MRD2, eyelid contour, and palpebral fissure has been previously described [13,14,15,16]. However, these methods rely on significant user and computer interaction after image acquisition and depend on an observer to identify the edges and facial features. Moreover, their accuracy and reproducibility are unsatisfactory because the partially automated measurement techniques depend on the user. With the development of AI technology, various deep learning methods are being applied in clinical medicine. Some researchers have attempted to quantitatively describe the eyelid contour on digital face images using the deep learning method. Shao et al. used an attention gate connection based on U-Net (Attention R2U-Net) to analyze eyelid morphology in thyroid-associated ophthalmopathy by measuring eyelid morphological parameters, including MRD1 (mm). However, these methods rely on computer interaction after image acquisition and compute the results separately, which limits their direct application in real-world clinical settings.

Lou et al. reported an image analysis technique using a deep learning method to compare surgical outcomes before and after blepharoplasty based on facial digital photographs taken separately [17].

The problem with previous studies is that they use the RGB image format. In reality, many people have dark irises, which is a barrier to direct clinical applications.

Additionally, several studies have used IR images for eye segmentation [18,19].

However, the main purpose of eye segmentation is gaze tracking to analyze human behavior, and it is not relevant to MRD1 measurements.

In other words, taking a photo and deep learning computation were separated in previous studies, which provided obstacles to the application of the technique in clinical practice. Therefore, we introduced a novel measurement method using deep learning that can simultaneously capture a photo and compute the MRD1 measurement results via IR image format.

To the best of our knowledge, this is the first hardware- and software-incorporated MRD1 measurement method that uses deep learning via IR image format. This is the first application of the iris and pupil center segmentation algorithm to objectively measure MRD1.

Also, the author acquired the images at the same time and in the same position to ensure reliability of the different image formats.

Depending on the angle between the patient’s eye and the IR camera, the MRD1 measurement value can vary significantly; therefore, keeping the lens of the camera and the patient’s eye as vertically parallel as possible is crucial to avoiding errors when using chin fixators.

In our study, observing the degree of difference between the RGB and IR images was crucial. Accurate detection of the pupil center is a prerequisite for precise MRD1 measurement. Detecting the pupil in an IR image is technically easy, given that the contours of the pupil are salient. Lighting conditions and reflections can significantly affect the pupil detection process in RGB images [20].

In our study, the Pearson correlation coefficient between MRD1_RGB ImageJ and MRD1_IR ImageJ was 0.754, indicating that the results were not identical. Furthermore, the distribution of the measured values was most similar between the deep learning method and IR ImageJ analysis.

In the Bland–Altman plot analysis, the mean difference between MRD1_DL and MRD1_IR ImageJ was the smallest at 0.0611, and the range of ΔLOA (upper limit of agreement–lower limit of agreement) values was smallest at 2.5162 compared to that of MRD1_manual and MRD1_RGB_analysis, for which the results were 0.4333 and 3.2438, respectively.

Considering that the four measurement groups are homogeneous on average in the ANOVA test, and the Pearson’s correlation value and degree of agreement are the highest for MRD1_DL and MRD1_IR_ImageJ, it can be concluded that this method, which utilizes an IR camera and a deep learning algorithm, is statistically superior to the manual method. Because we identified that the novel method developed by the author has statistical accuracy, considering the simplicity, cost-effectiveness, and time efficiency of the procedure, this novel method can be used directly in clinical settings.

This study had several limitations, which are as follows.

First, the data obtained from the RGB and IR images did not match perfectly regarding time and position, producing measurement errors.

Second, the lack of an actual absolute value for MRD1 makes it challenging to rely on the gold standard as a reference, thus diminishing the statistical significance of this study. Therefore, more precise and accurate methods must be developed in the near future.

Finally, the data obtained from this study were restricted to MRD1, and other periorbital measurements, such as MRD2 and palpebral fissure height, will be studied in the future.

## 5. Conclusions

In this study, we found that the newly introduced MRD1 measurement method using IR image-based deep learning does not show statistically significant differences from the existing manual method and gold-standard measurements. We also found that the method using deep learning and the method using IR have the highest agreement and are faster than existing methods. In conclusion, these findings suggest that the newly introduced method can serve as a promising alternative for measuring MRD1 with statistical accuracy.

## Figures and Tables

**Table 1:** Descriptive statistics for four different MRD1 measurement methods.

Descriptive Statistics for Four Different MRD1 Measurement Methods								
MRD1(mm)								
	N	Mean	Std. Deviation	Std. Error	95% Confidence Interval for Mean		Minimum	Maximum
					Lower Bound	Upper Bound		
MRD1_manual	56	2.6404	1.03859	0.13879	2.3622	2.9185	0.83	5.07
MRD1_DL	56	2.8450	1.07324	0.14342	2.5576	3.1324	0.74	5.27
MRD1_IR_ ImageJ	56	2.7839	1.07560	0.14373	2.4959	3.0720	1.09	5.70
MRD1_RGB_ ImageJ	56	3.0737	0.94669	0.12651	2.8201	3.3272	1.52	5.68
Total	224	2.8357	1.03971	0.06947	2.6988	2.9726	0.74	5.70

**Table 2:** One-way analysis of variance test between four different methods (*p* = 0.168).

ANOVA					
MRD1 (mm)					
	Sum of Squares	df	Mean Square	F	Sig.
Between Groups	5.463	3	1.821	1.700	0.168
Within Groups	235.601	220	1.071		
Total	241.064	223			

**Table 3:** Post hoc analysis (Scheffe) for four different MRD1 measurement methods. There exists no statistically significant difference among the four groups.

Post hoc Analysis for Four Different MRD1 Measurement Methods.						
Dependent Variable: MRD1 (mm)						
Scheffe						
(I) Methods	(J) Methods	Mean Difference (I-J)	Std. Error	Sig.	95% Confidence Interval	
					Lower Bound	Upper Bound
MRD1_manual	MRD1_DL	−0.20464	0.19557	0.778	−0.7556	0.3463
	MRD1_IR_ImageJ	−0.14355	0.19557	0.910	−0.6945	0.4074
	MRD1_RGB_ImageJ	−0.43330	0.19557	0.182	−0.9843	0.1177
MRD1_DL	MRD1_manual	0.20464	0.19557	0.778	−0.3463	0.7556
	MRD1_IR_ImageJ	0.06109	0.19557	0.992	−0.4899	0.6121
	MRD1_RGB_ImageJ	−0.22866	0.19557	0.714	−0.7796	0.3223
MRD1_IR_ImageJ	MRD1_manual	0.14355	0.19557	0.910	−0.4074	0.6945
	MRD1_DL	−0.06109	0.19557	0.992	−0.6121	0.4899
	MRD1_RGB_ImageJ	−0.28975	0.19557	0.534	−0.8407	0.2612
MRD1_RGB_ImageJ	MRD1_manual	0.43330	0.19557	0.182	−0.1177	0.9843
	MRD1_DL	0.22866	0.19557	0.714	−0.3223	0.7796
	MRD1_IR_ImageJ	0.28975	0.19557	0.534	−0.2612	0.8407

**Table 4:** Pearson correlation analysis for four different MRD1 measurement methods.

Pearson Correlations Analysis for Different Measurement Methods					
		MRD1_Manual	MRD1_DL	MRD1_IR_ImageJ	MRD1_RGB_ImageJ
**MRD1_manual**	Pearson Correlation	1	0.473 **	0.603 **	0.656 **
	Sig. (2-tailed)		0.000	0.000	0.000
	N	56	56	56	56
**MRD1_DL**	Pearson Correlation	0.473 **	1	0.822 **	0.543 **
	Sig. (2-tailed)	0.000		0.000	0.000
	N	56	56	56	56
**MRD1_IR_ImageJ**	Pearson Correlation	0.603 **	0.822 **	1	0.720 **
	Sig. (2-tailed)	0.000	0.000		0.000
	N	56	56	56	56
**MRD1_RGB_ImageJ**	Pearson Correlation	0.656 **	0.543 **	0.720 **	1
	Sig. (2-tailed)	0.000	0.000	0.000	
	N	56	56	56	56

Note that the significance of *p* < 0.005 is indicated by **.

## Data Availability

The data presented in this study are available upon request from the corresponding author. The data are not publicly available due to patients’ privacy.

## References

[1] Boboridis K., Assi A., Indar A., Bunce C., Tyers A. (2001). Repeatability and reproducibility of upper eyelid measurements. Br. J. Ophthalmol..

[2] Nemet A.Y. (2015). Accuracy of marginal reflex distance measurements in eyelid surgery. J. Craniofacial Surg..

[3] Coombes A.G., Sethi C.S., Kirkpatrick W.N., Waterhouse N., Kelly M.H., Joshi N. (2007). A standardized digital photography system with computerized eyelid measurement analysis. Plast. Reconstr. Surg..

[4] Chun Y.S., Park H.H., Park I.K., Moon N.J., Park S.J., Lee J.K. (2017). Topographic analysis of eyelid position using digital image processing software. Acta Ophthalmol..

[5] Liu N., Liang G., Li L., Zhou H., Zhang L., Song X. (2021). An eyelid parameters auto-measuring method based on 3D scanning. Displays.

[6] Song X., Tong W., Lei C., Huang J., Fan X., Zhai G., Zhou H. (2021). A clinical decision model based on machine learning for ptosis. BMC Ophthalmol..

[7] Sahoo M., Ghorai S., Pal S., Mitra M. (2022). A Multi-Layer stacked ensemble classifier model for improved classification accuracy of Maculopathy gradation. Displays.

[8] Lei C., Qu M., Sun H., Huang J., Huang J., Song X., Zhai G., Zhou H. (2023). Facial expression of patients with Graves’ orbitopathy. J. Endocrinol. Investig..

[9] Luo R., Ge Y., Hu Z., Liang D., Li Z.-C. (2021). DeepPhase: Learning phase contrast signal from dual energy X-ray absorption images. Displays.

[10] Mariakakis A., Baudin J., Whitmire E., Mehta V., Banks M.A., Law A., Mcgrath L., Patel S.N. (2017). PupilScreen: Using smartphones to assess traumatic brain injury. Proc. ACM Interact. Mob. Wearable Ubiquitous Technol..

[11] McAnany J.J., Smith B.M., Garland A., Kagen S.L. (2018). iPhone-based pupillometry: A novel approach for assessing the pupillary light reflex. Optom. Vis. Sci..

[12] Chaudhary A.K., Kothari R., Acharya M., Dangi S., Nair N., Bailey R., Kanan C., Diaz G., Pelz J.B. Ritnet: Real-time semantic segmentation of the eye for gaze tracking. Proceedings of the 2019 IEEE/CVF International Conference on Computer Vision Workshop (ICCVW).

[13] Bodnar Z.M., Neimkin M., Holds J.B. (2016). Automated ptosis measurements from facial photographs. JAMA Ophthalmol..

[14] Burmann T.G., Valiatti F.B., Correa Z.M., Bayer M., Marcon Í. (2008). Margin reflex distance measure by computerized image processing in rigid contact lens wearers. Arq. Bras. De Oftalmol..

[15] Cruz A., Lucchezi M.C. (1999). Quantification of palpebral fissure shape in severe congenital blepharoptosis. Ophthalmic Plast. Reconstr. Surg..

[16] Cruz A.A., Coelho R.P., Baccega A., Lucchezi M.C., Souza A.D., Ruiz E.E. (1998). Digital image processing measurement of the upper eyelid contour in Graves disease and congenital blepharoptosis. Ophthalmology.

[17] Shao J., Huang X., Gao T., Cao J., Wang Y., Zhang Q., Lou L., Ye J. (2023). Deep learning-based image analysis of eyelid morphology in thyroid-associated ophthalmopathy. Quant. Imaging Med. Surg..

[18] Solyman O., Abushanab M.M.I., Carey A.R., Henderson A.D. (2022). Pilot study of smartphone infrared pupillography and pupillometry. Clin. Ophthalmol..

[19] Cherif Z.R., Nait-Ali A., Motsch J., Krebs M. An adaptive calibration of an infrared light device used for gaze tracking. Proceedings of the IMTC/2002. Proceedings of the 19th IEEE Instrumentation and Measurement Technology Conference (IEEE Cat. No. 00CH37276).

[20] Lu C., Chakravarthula P., Liu K., Liu X., Li S., Fuchs H. Neural 3D Gaze: 3D Pupil Localization and Gaze Tracking based on Anatomical Eye Model and Neural Refraction Correction. Proceedings of the 2022 IEEE International Symposium on Mixed and Augmented Reality (ISMAR).

